# Expression and prognostic significance of cathepsin D in acute myeloid leukemia

**DOI:** 10.1097/MD.0000000000045313

**Published:** 2025-10-17

**Authors:** Hui Han, Yunxiu Huang, Jinye Xie, Jiahui Liu, Weijia Wang

**Affiliations:** a Department of Laboratory Medicine, Zhongshan City People’s Hospital, Zhongshan, China.

**Keywords:** acute myeloid leukemia, biomarker, CTSD, prognosis

## Abstract

This study investigates the expression level of cathepsin D (CTSD) in the serum of patients with acute myeloid leukemia (AML) and its clinical significance and evaluates its potential value as a prognostic biomarker for AML. Bioinformatics analysis was performed to examine the expression profile and prognostic correlation of CTSD in AML. A total of 63 newly diagnosed AML patients and 50 healthy controls were enrolled in validation. Serum CTSD levels were measured using enzyme-linked immunosorbent assay, and their associations with clinical characteristics, treatment response, and prognosis were analyzed. Bioinformatics analysis showed CTSD may be a potential factor for poor prognosis in AML patients. Validation through sample analysis confirmed that the serum CTSD level in AML patients (400.00 ± 240.00 pg/mL) was significantly higher than in healthy controls(*P* < .001). CTSD expression was positively correlated with peripheral white blood cell count and bone marrow blast percentage. Patients who achieved complete remission after chemotherapy had significantly lower CTSD levels than those in the non-remission and partial remission groups. Survival analysis revealed that patients with high CTSD expression had significantly shorter disease-free survival (*P* = .021) and overall survival (*P* = .026). Risk stratification showed the level of CTSD was lower in favorable group than that in intermediate and poor groups. Multivariate regression analysis showed that CTSD was a significant independent risk factor in both the overall survival and disease-free survival models, with a hazard ratio 1.002. The expression level of CTSD in peripheral serum is significantly elevated in AML patients and is closely associated with disease progression and poor prognosis. CTSD may serve as a potential biomarker for AML prognostic assessment.

## 1. Introduction

Acute myeloid leukemia (AML) is a malignant tumor originating from hematopoietic stem/progenitor cells, with an increasing incidence trend. AML is the most common cause of leukemia-related deaths in adults, with a 5-year overall survival rate of approximately 24%.^[1]^ Although advances in chemotherapy regimens, targeted therapies, and hematopoietic stem cell transplantation in recent years have significantly improved remission rates in some patients, disease relapse and drug resistance remain the primary causes of treatment failure.^[2]^ Studies have shown that multi-drug resistance is one of the key factors leading to AML chemotherapy failure.^[3,4]^ Therefore, exploring new molecular markers to optimize AML diagnosis, prognostic assessment, and individualized treatment strategies holds significant clinical value.

Cathepsin D (CTSD) is an aspartic protease mainly found in lysosomes. It participates in the degradation of intracellular proteins and the extracellular matrix, thereby promoting tumor cell motility and enabling tumor cells to traverse the extracellular matrix and vascular walls into the bloodstream. Over-expression of CTSD not only stimulates tumor initiation and metastasis but also plays a crucial role in various stages of tumor progression.^[5]^ Recent studies have found that CTSD plays an important role in multiple malignancies (such as breast cancer, prostate cancer, and gliomas) as well as inflammatory diseases, including mechanisms that encourage tumor cell growth, spread, and metastasis, while preventing apoptosis, thus contributing to disease progression.^[6–8]^ However, the expression pattern and clinical significance of CTSD in AML remain unclear. In-depth research on CTSD expression characteristics in AML and its association with disease prognosis may provide new targets for AML diagnosis and treatment.

In this study, we investigated the serum expression level of CTSD in AML patients and its correlations with clinical features, treatment response, and prognosis through integrated bioinformatics analysis and clinical sample validation. Based on The Cancer Genome Atlas (TCGA) database, we initially assessed the expression profile and prognostic significance of CTSD. Serum CTSD levels were then measured via enzyme-linked immunosorbent assay (ELISA) in newly diagnosed AML patients and healthy controls. This study aims to determine whether CTSD can serve as a potential diagnostic and prognostic marker for AML, thereby providing a theoretical basis for optimizing precision treatment of AML.

## 2. Materials and methods

### 2.1. Bioinformatics analysis

Bioinformatics analyses were conducted to characterize CTSD expression in AML and its prognostic relevance using TCGA-LAML data (https://portal.gdc.cancer.gov). RNA-seq read counts processed by the STAR-counts pipeline (STAR v2.7.10b, GRCh38) and matched clinical data, including age, sex, French–American–British (FAB) subtype, 2022 European LeukemiaNet (ELN) risk, overall survival (OS) time/status, disease-free survival (DFS) time, which were obtained. newly diagnosed cases with complete RNA-seq (mapping rate ≥ 90%, duplication rate ≤ 30%) and full follow-up were included; relapsed/refractory cases and samples with key clinical omissions were excluded, yielding 139 patients (18 M3, all PML/RARA-positive; 121 non-M3 spanning FAB M1–M7/unclassified). Counts were converted to transcripts per million (TPM) and log 2-transformed (log 2[TPM + 1]); genes with TPM ≥ 1 in <50% of samples were filtered out. Analyses were performed in R v4.0.3 using validated packages: CTSD expression (log 2[TPM + 1]) was summarized across AML subtypes (ggplot2 v3.4.4); prognostic associations were evaluated by Kaplan–Meier analysis comparing high (≥median) versus low (<median) CTSD groups for OS and DFS with log-rank testing (survival v3.5.5; survminer v0.4.9); and predictive performance was assessed by time-dependent receiver operating characteristic curves for 1-, 3-, and 5-year OS with area under the curves (pROC v1.18.4). Statistical significance was set at 2-sided *P* < .05.

### 2.2. Patient samples

This study included 63 newly diagnosed AML patients and 50 healthy controls recruited at Zhongshan People’s Hospital from January 2021 to December 2023. All AML diagnoses were confirmed through bone marrow morphology, cytochemical staining, flow cytometry, and genetic testing. Disease classification followed the FAB classification system. All patients were diagnosed according to World Health Organization blood tumor classification standard^[9]^ and the latest international consensus classification of myeloid neoplasms and acute leukemia.^[10]^ Patients with hepatic or renal dysfunction, malnutrition, or diabetes, as well as those unable to tolerate chemotherapy drugs, were excluded. Informed consent was obtained from all participants, and the study was approved by the Ethics Committee of Zhongshan People’s Hospital.

The baseline (pretreatment) group consisted of AML patients prior to therapy. Initial treatment regimens varied depending on cytogenetic and molecular biology results, and standard-dose chemotherapy was administered. Induction therapy regimens for AML patients were as follows: non-M3 patients received standard daunorubicin and cytarabine, idarubicin and cytarabine, pirarubicin and cytarabine, or homoharringtonine and cytarabine; M3 patients received differentiation therapy with all-trans retinoic acid or arsenic trioxide. After 2 cycles of treatment, therapeutic outcomes were evaluated according to 2022 ELN recommendations,^[11]^ and classified as complete remission (CR), partial remission (PR), or no response (NR).

Clinical data were collected for each patient, including gender, age, proportion of blast cells in bone marrow and peripheral blood, lactate dehydrogenase levels, white blood cell count, red blood cell count, hemoglobin, and platelet count. Genetic information was also collected, including fusion genes such as PML/RARA and BCR/ABL, as well as mutation sites related to risk stratification, such as Fms-like tyrosine kinase 3 internal tandem duplication mutations, histone methyltransferase enhancer of zeste homolog 2, CCAAT Enhancer Binding Protein Alpha, tumor protein p53, and Runt-related transcription factor 1, by real-time quantitative reverse transcription-polymerase chain reaction. All laboratory test results were obtained through the hospital’s laboratory information system (Chuanghui system).

### 2.3. Serological testing

The first blood samples were collected from patients upon hospital admission, and the second set was collected after 2 cycles of chemotherapy. Serum samples were stored at −70°C. The level of CTSD in peripheral blood (PB) was measured using ELISA kits (JL12469) from the same batch (Shanghai Jianglai Biotechnology Co., Ltd., Shanghai, China). Prior to analysis, serum samples and reagents were equilibrated at room temperature (25°C) for 60 minutes, with standard wells and sample wells set up accordingly. The assay was then conducted according to the manufacturer’s instructions. Optical density values at 450 nm were measured using a Thermo Scientific Fluoroskan FC microplate reader (Catalog No. 51119080, Thermo Fisher Scientific, Waltham, MA), and a standard curve was used to calculate serum CTSD concentrations. Instruments used also included a 37°C constant temperature incubator (Model: HH-W600, OLABO Technology Co., Ltd., Jinan, Shandong, China).

### 2.4. Follow-up

Follow-up was conducted through medical record reviews or phone calls, with a cutoff date of February 2024. The median follow-up duration was 17 months (range: 1–36 months). OS was defined as the time from diagnosis to death from any cause or last follow-up. DFS was defined as the time from CR to relapse. Because M3 AML is uniquely treated with all-trans retinoic acid and arsenic trioxide-based targeted therapy, which achieved favorable clinical outcomes and prognoses, DFS and OS were assessed only in non-M3 patients.

### 2.5. Statistical analysis

Comparisons between 2 or more groups were performed using the chi-square test, and continuous data were presented as medians (ranges) by SPSS software, version 22.0.0 (International Business Machine, Armonk, NY). Survival analysis was performed using the survival package in R (v4.2.1) for Kaplan–Meier curve plotting and log-rank testing. Correlation analysis was carried out with GraphPad Prism 8.0 software version (GraphPad Software, San Diego, CA) to calculate Spearman correlation coefficients. A *P*-value of <.05 was considered statistically significant. For univariate and multivariate analysis, the Cox risk model was used. Statistical significance was set at *P* < .05.

## 3. Results

### 3.1. TCGA database indicates CTSD may be a potential factor for poor prognosis in AML patients

**Figure 1.**
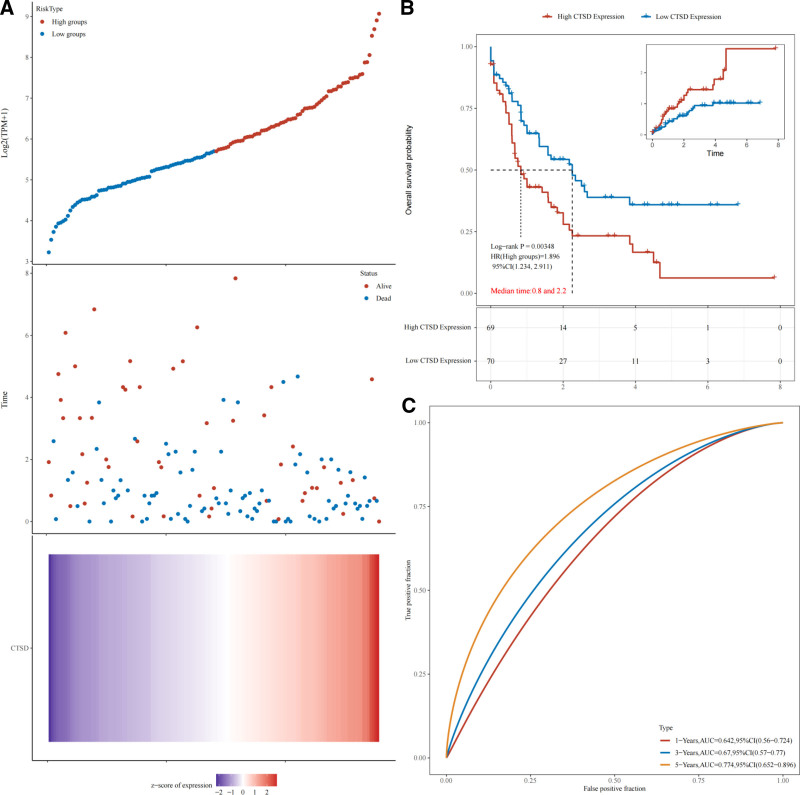
Prognostic analysis of CTSD expression in TCGA-LAML dataset. (A) Association between CTSD gene expression and survival characteristics in TCGA dataset. The upper scatter plot shows CTSD expression levels (Log 2 (TPM + 1)), colored by expression groups (high in red, low in blue). The middle scatter plot presents survival time and survival status (alive or dead) distributions across samples. The bottom plot is a heatmap representing CTSD expression intensity, indicated by *Z*-score. (B) Kaplan–Meier (KM) survival curve analysis of CTSD expression groups. The survival differences were evaluated using the log-rank test (*P* = .00348). The hazard ratio (HR) for the high-expression group compared to the low-expression group is 1.896 (95% CI: 1.234–2.911), indicating CTSD as a risk factor. Median survival times are 0.8 years for the high-expression group and 2.2 years for the low-expression group. The inset graph depicts the cumulative probability of adverse outcomes over time. (C) Time-dependent ROC curves evaluating the predictive accuracy of CTSD expression for survival at 1, 3, and 5 years, with corresponding AUC values of 0.642 (95% CI: 0.560–0.724), 0.670 (95% CI: 0.570–0.770), and 0.774 (95% CI: 0.652–0.896), respectively. Higher AUC values indicate stronger prognostic capabilities. AML = acute myeloid leukemia, AUC = area under the curves, CTSD = cathepsin D, CI = confidence interval, HR = hazard ratio, TCGA = The Cancer Genome Atlas.

Expression profile data were collected from TCGA database. Samples lacking complete sequencing or clinical data were excluded, resulting in 139 samples for analysis. Survival differences between high- and low-expression groups were evaluated with Kaplan–Meier (KM) analysis. The results showed that the high-expression group had shorter survival times than the low-expression group, with statistically significant differences between the 2 (*P* < .001) (Fig. 1A, B). CTSD gene expression demonstrates progressively stronger prognostic predictive ability over time, showing optimal accuracy for 5-year survival prediction (area under the curve = 0.774), which is significantly better than its 1-year and 3-year predictive performance (Fig. 1C).

### 3.2. Clinical characteristics of study subjects

This study included 63 newly diagnosed AML patients and 50 healthy controls. Among the AML patients, 12 were classified as M3 type and 51 as non-M3 type. The characteristics of all participants are summarized in Table 1.

**Table 1:** Demographic and clinical characteristics of subjects.

Clinical parameter	AML (n = 63)	Healthy controls (n = 50)	*P*
Age (yr)	44.43 ± 16.57	52.58 ± 12.37	.325
Gender (man/woman)	34/29	28/22	–
Bone marrow blasts (%)	61.40 ± 23.82	–	
Peripheral blood blasts (%)	48.29 ± 30.30	–	
LDH (U/L)	560.8 ± 451.9	169.25 ± 67.45	<.001
WBC (×10^9^/L)	30.54 ± 24.96	6.21 ± 1.38	<.001
RBC (×10^12^/L)	3.02 ± 0.97	4.94 ± 0.62	<.001
HGB (g/L)	85.32 ± 27.81	124.05 ± 27.84	<.001
PLT (×10^12^/L)	76.19 ± 60.32	208.13 ± 67.08	<.001

AML = acute myeloid leukemia, BM = bone marrow, HGB = hemoglobin, PM = peripheral blood, LDH = lactate dehydrogenase, RBC = red blood cell, WBC = white blood cell.

### 3.3. Expression pattern and prognostic value of CTSD in AML

**Figure 2.**
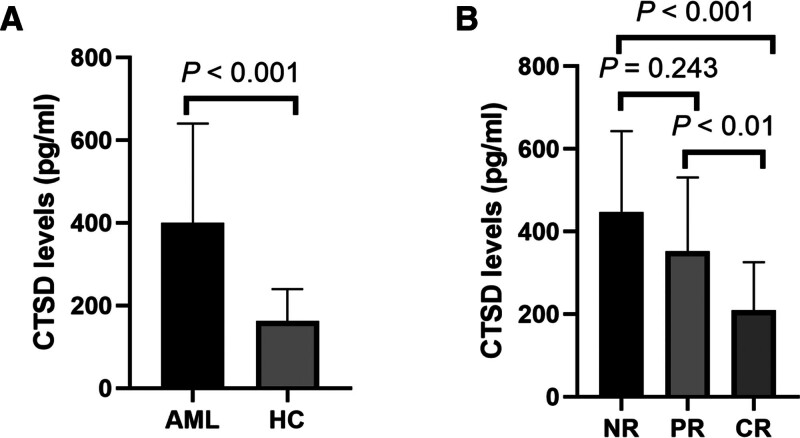
The concentration of CTSD in myeloid leukemia groups and healthy controls. (A) The level of CTSD in the initial diagnosed myeloid leukemia group (400.0 ± 240.0 pg/mL, n = 63) and healthy controls (162.5 ± 77.31 pg/mL, n = 50). (B) The level of CTSD in the subgroups of first chemotherapy. NR (447.8 ± 195.0 pg/mL, n = 11), PR (351.3 ± 179.7 pg/mL, n = 14), CR (209.0 ± 116.6 pg/mL, n = 38). CR = complete remission, CTSD = cathepsin D, NR = no response, PR = partial remission.

To verify the serum levels of CTSD in each experimental group, we measured CTSD levels in 63 newly diagnosed AML patients and 50 healthy controls using ELISA. The results showed that serum CTSD levels in the AML group (400.00 ± 240.00 pg/mL) were significantly higher than those in the control group (176.50 ± 132.10 pg/mL) (*P* < .001) (Fig. 2A).

After 2 cycles of standard chemotherapy, 38 AML patients achieved CR, 14 achieved PR, and 11 showed NR. CTSD expression in the CR group (209.0 ± 116.6 pg/mL) was significantly lower than in the NR group (447.8 ± 195.0 pg/mL, n = 11) and the PR group (351.3 ± 179.7 pg/mL, n = 14) (*P* < .05). However, there was no statistically significant difference between the PR and NR groups (Fig. 2B).

### 3.4. Correlation between serum CTSD levels and clinical features

**Figure 3.**
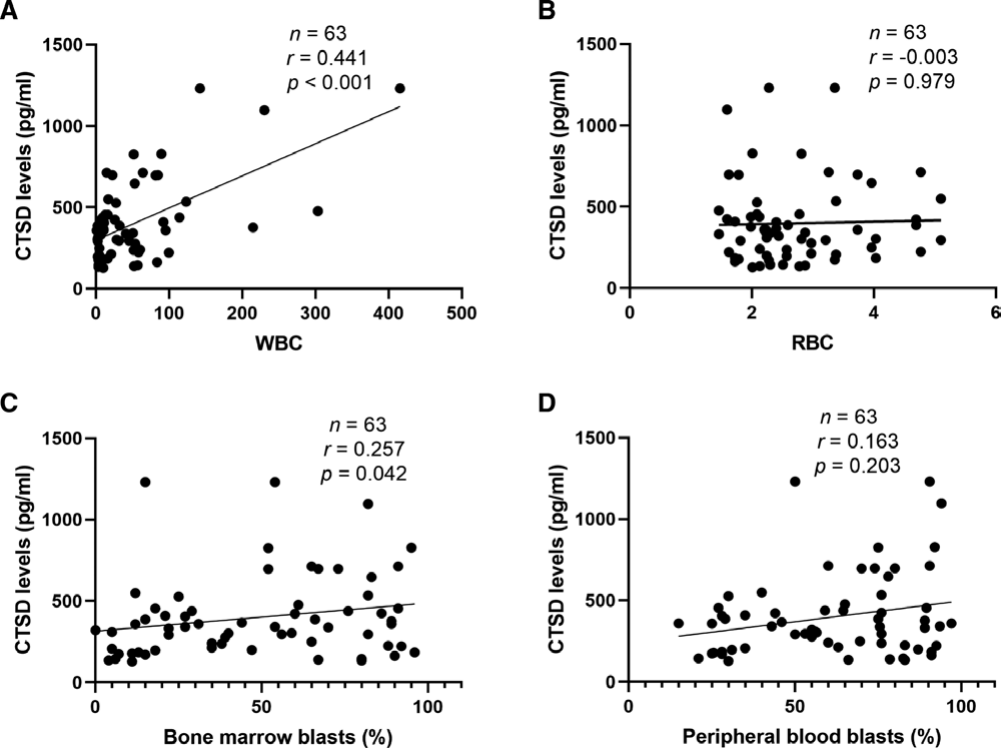
Correlations of CTSD levels with WBC, RBC, blasts in BM and blasts in PB. (A–D) Correlations of plasma CTSD levels with WBC count in peripheral blood (A), RBC count in peripheral blood (B), blasts in bone marrow (C), and blasts in peripheral blood (D) were analyzed. Spearman correlation coefficient ® and *P*-value of linear regression are shown in each panel. BM = bone marrow, PB = peripheral blood, RBC = red blood cell, WBC = white blood cell.

Spearman correlation analysis showed that serum CTSD levels were correlated with white blood cell (WBC) counts in peripheral blood and the proportion of blasts in bone marrow. However, no specific correlation was found with red blood cell counts or the proportion of blasts in peripheral blood. The results are shown in Figure 3.

### 3.5. FAB classification and CTSD expression in AML subtypes

Using ELISA to measure CTSD expression levels, significant differences were found between AML subtypes and the control group (*P* < .01) (Table 2). Among them, the M5 subtype exhibited the highest expression level of CTSD, while the subtype with unclear classification showed the lowest expression level. However, no statistically significant differences were observed among the various AML subtypes. The results are shown in Table 2.

**Table 2:** Expressions of CTSD in AML and control groups.

Group	Typing	N	CTSD (pg/mL) median (range)
AML	M1	4	446.7 (221.4–796.2)*
	M2	20	362.9 (162.7–1018.0)**
	M3	12	396.0 (128.6–1097.0)**
	M4	12	456.0 (144.6–1231.0)*
	M5	8	493.5 (174.3–828.3)*
	No-known type	7	316.2 (135.1–534.1)**
Healthy control		50	176.5 (38.82–428.3)

AML = acute myeloid leukemia, CTSD = cathepsin D.

* *P* < .001.

** *P* < .01 versus control group.

### 3.6. Association between CTSD expression and prognosis in AML patients

**Figure 4.**
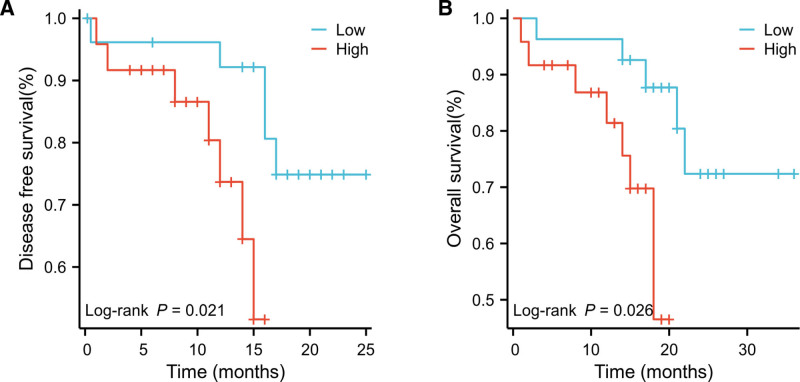
Disease-free survival (DFS) and overall survival (OS) curves of non-M3 AML patients with different CTSD. (A) High CTSD expression patients had significantly shorter disease-free survival than low CTSD patients (*P* = .021). (B) The Kaplan–Meier curves showed that AML patients with high CTSD expression had significantly shorter overall survival than low CTSD patients (*P* = .026).

AML patients were divided into high- and low-expression groups based on CTSD levels, with the top 50% classified as the high-expression group and the bottom 50% as the low-expression group. Survival analysis was conducted using the Kaplan–Meier method. The results showed that AML patients with high CTSD expression had significantly shorter DFS and OS compared to the low-expression group (*P* < .05, Fig. 4). High expression of CTSD in serum samples was significantly associated with poor prognosis in AML patients.

**Figure 5.**
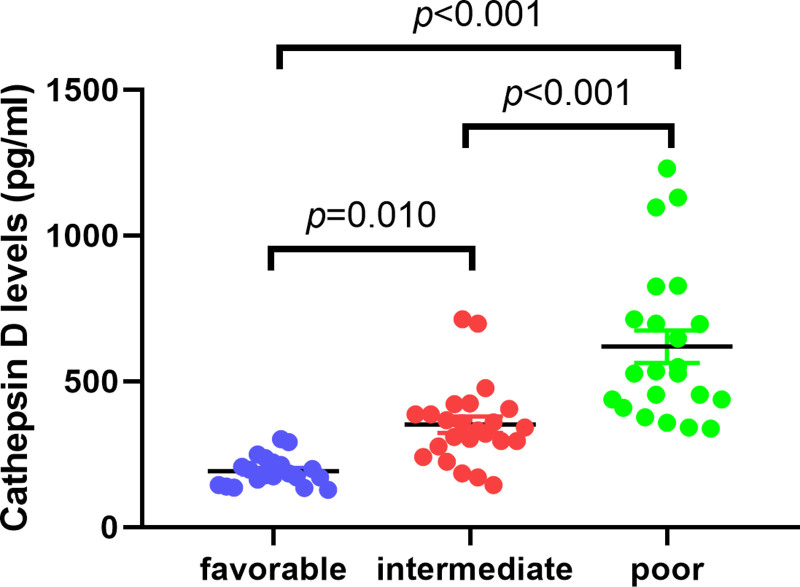
The expression of CTSD in favorable, intermediate and poor groups. The expression of CTSD in 3 groups was significantly different (*P* < .01), poor group had higher cathepsin D expression than favorable and intermediate groups (*P* < .01).

According to the 2022 European LeukemiaNet^[11]^ classification, which incorporates fusion genes, genetic mutations, and cytogenetic abnormalities, 63 AML patients were stratified into favorable, intermediate, and adverse risk groups. It was found that patients in the favorable prognosis group had significantly lower CTSD concentrations than those in the intermediate and adverse groups (Fig. 5). Moreover, as cytogenetic risk increased, CTSD levels showed a marked upward trend. A univariate analysis was conducted to identify the primary factors influencing patient survival, including age, sex, the proportion of bone marrow and PB blast cells, WBC count at initial diagnosis, and risk stratification. In the multivariate analysis factors with *P* values <.05 were considered, affecting AML patient survival. Multivariate Cox proportional hazards regression analysis demonstrated that after adjusting for the ELN risk stratification, CTSD remained an independent adverse prognostic factor for DFS and OS (hazard ratio = 1.002), as indicated in Table 3.

**Table 3:** Univariate and multivariate analysis of factors influencing disease-free survival and overall survival.

Factor	Univariate analysis		Multivariate analysis	
	HR (95% CI)	*P* value	HR (95% CI)	*P* value
DFS				
CTSD (≥341.62 pg/mL)	1.01 (1.00–1.02)	.008*	1.002 (1.001–1.003)	.005*
Sex (female)	1.15 (0.99–1.03)	.312		
Age (≥60 yr)	1.01 (1.00–1.02)	.008*	1.00 (0.98–1.03)	.685
Blasts in BM (≥60%)	1.00 (0.99–1.01)	.832		
Blasts in PB (≥64.5%)	0.97 (0.99–1.01)	.721		
WBC (≥30 × 10^9^/L)	1.00 (0.99–1.01)	.912		
Risk stratification				
Intermediate versus low	1.29 (0.75–2.23)	.355		
High versus low	2.16 (1.24–3.78)	.007**	1.36 (0.65–2.84)	.412
OS				
CTSD (≥341.62 pg/mL)	1.01 (1.00–1.02)	.005*	1.002 (1.000–1.003)	.041**
Sex (male)	1.23 (0.85–1.78)	.271		
Age (≥60 yr)	1.02 (1.00–1.04)	.043**	1.00 (0.97–1.03)	.839
Blasts in BM (≥60%)	1.01 (1.00–1.02)	.789		
Blasts in PB (≥64.5%)	1.00 (0.99–1.01)	.654		
WBC (≥30 × 10^9^/L)	1.00 (0.99–1.01)	.874		
Risk stratification				
Intermediate versus low	1.21 (0.66–2.22)	.537		
High versus low	2.59 (1.45–4.62)	.001*	1.3 (0.4–4.3)	.694

Characteristics of the 63 AML patients before therapy used in this study.

AML = acute myeloid leukemia, BM = bone marrow, CI = confidence interval, CTSD = cathepsin D, DFS = disease-free survival, HR = hazard ratio, OS = overall survival, PB = peripheral blood, WBC = white blood cell.

*P* < .05, indicating a statistical significant difference.

* *P* < .01.

** *P* < .05.

## 4. Discussion

Acute myeloid leukemia is a highly heterogeneous malignant hematologic disease. Despite significant progress in chemotherapy, targeted therapies, and hematopoietic stem cell transplantation in recent years, relapse and mortality rates remain high. Disease relapse and drug resistance continue to be major causes of treatment failure.^[12]^ Therefore, identifying new molecular targets is of great clinical importance for the diagnosis and prognostic assessment of AML. This study, through a combination of bioinformatics analysis and clinical sample validation, explored the expression characteristics and clinical significance of serum CTSD in AML patients. Our findings suggest that CTSD is significantly over-expressed in the serum of AML patients and that its expression is closely related to disease progression, treatment response, and prognosis, indicating that CTSD may serve as a potential diagnostic and prognostic biomarker for AML.

ELISA results showed that serum CTSD levels were significantly elevated in newly diagnosed AML patients compared to healthy individuals (*P* < .001). This elevation was consistently observed across all AML FAB subtypes, with the most pronounced increase noted in M5 subtypes, suggesting a potential association with monocytic differentiation. This observation aligns with previous reports of CTSD over-expression in AML^[13]^ and solid tumors such as breast cancer and hepatocellular carcinoma.^[8,14,15]^ Recent studies^[13]^ have shown CTSD promotes acute myeloid leukemia progression, knocking down CTSD in leukemia cells curtails cell proliferation and antiapoptotic effects in vitro, and eases AML development in vivo, suggesting that CTSD may contribute to AML pathogenesis by promoting tumor cell proliferation, invasion, and metastasis.

Research has shown that CTSD is a lysosomal aspartic protease with dual functions, promoting apoptosis via its mature form inside cells and cell proliferation via its pro-enzyme form outside cells. This dual functionality suggests that CTSD could play a significant role in the progression of AML by influencing cell fate decisions, thereby contributing to the disease’s aggressive nature.^[16]^ Although no statistically significant differences in CTSD expression were found among AML FAB subtypes, expression levels were consistently higher than in controls, indicating CTSD may serve as a broad-spectrum diagnostic marker for AML, independent of morphological classification.

Our study also found that CTSD expression levels positively correlated with peripheral white blood cell counts and the proportion of bone marrow blasts, suggesting a potential link between CTSD and AML tumor burden. This further supports the role of CTSD in leukemic cell proliferation. Additionally, post-chemotherapy serum CTSD levels in patients achieving complete remission were significantly lower than in those with no response or partial remission (*P* < .05). This indicates that dynamic changes in CTSD expression can reflect disease progression. As patients improved, CTSD levels declined, suggesting a potential role in AML chemotherapy resistance. However, the exact mechanisms remain to be elucidated. For instance, it is worth investigating whether CTSD influences chemotherapy drug distribution or metabolism by regulating lysosomal function, or whether it promotes cell survival by activating downstream signaling pathways.^[17]^

Analysis of the TCGA databases revealed significantly high-expression CTSD group had shorter survival times than low-expression group, a finding that was further validated in clinical samples. Survival analysis revealed that AML patients in the high CTSD expression group had significantly shorter disease-free survival and overall survival compared to those in the low-expression group (*P* < .05), consistent with TCGA database results. More importantly, according to the 2022 ELN risk stratification criteria, patients in the favorable prognosis group had significantly lower CTSD levels than those in the intermediate and adverse groups. Furthermore, CTSD expression increased progressively with higher cytogenetic risk. This suggests that CTSD may serve as a prognostic factor independent of conventional genetic markers and provide supplementary information for AML risk stratification. A multivariate Cox regression analysis to adjust for established prognostic factors, including age, sex, the proportion of bone marrow and PB blast cells, WBC count at initial diagnosis, and risk stratification, high expression of CTSD remained an independent adverse prognostic factor for shortened DFS and OS. This indicates that the prognostic value of CTSD provides supplementary information to the existing risk stratification system.

Although CTSD’s potential importance in AML has been initially identified, we recognize that this study has certain limitations. First, the sample size, though substantial, remains a limitation. Therefore, to firmly establish CTSD as a reliable prognostic biomarker, our results necessitate further validation in larger, prospective, multi-center studies and, most importantly, in independent external patient cohorts. Some subgroup analyses in this study (such as NR group and certain rare FAB subtypes) may have limited statistical power due to small sample sizes. Therefore, the interpretation of these subgroup results should be made with caution and further validated in larger prospective cohorts. Second, potential heterogeneity in treatment regimens and subclassification of AML patients may have introduced confounding effects, despite our efforts to control for major clinical variables. Third, while we identified significant clinical correlations, mechanistic and functional studies are needed to elucidate the precise biological role of CTSD in AML pathogenesis. Finally, we will test speculative mechanistic hypotheses, such as the involvement of CTSD in autophagy, leukemic stem cell maintenance, or immune microenvironment modulation, to deepen the biological and translational understanding of CTSD in AML.

To our knowledge, this is the first study combining clinical serum analysis and transcriptomic profiling to investigate CTSD expression in AML. CTSD levels are closely associated with disease burden, treatment response, and prognosis. CTSD may serve as a novel molecular biomarker for the diagnosis and prognostic evaluation of AML and also provide a potential therapeutic target for overcoming chemotherapy resistance. Future studies with larger sample sizes and mechanistic investigations are needed to facilitate the clinical application of CTSD in the precision diagnosis and treatment of AML.

## Author contributions

**Data curation:** Yunxiu Huang.

**Funding acquisition:** Weijia Wang.

**Methodology:** Jiahui Liu.

**Software:** Jinye Xie.

**Writing – original draft:** Hui Han.

**Writing – review & editing:** Hui Han.
